# Wide distribution and ancient evolutionary history of simian foamy viruses in New World primates

**DOI:** 10.1186/s12977-015-0214-0

**Published:** 2015-10-29

**Authors:** Bruno M. Ghersi, Hongwei Jia, Pakorn Aiewsakun, Aris Katzourakis, Patricia Mendoza, Daniel G. Bausch, Matthew R. Kasper, Joel M. Montgomery, William M. Switzer

**Affiliations:** U.S. Naval Medical Research Unit No. 6, Lima, Peru; Laboratory Branch, Division of HIV/AIDS Prevention, National Center for HIV/AIDS, Viral Hepatitis, STD, and TB Prevention, Centers for Disease Control and Prevention, 1600 Clifton Rd., MS G-45, Atlanta, GA 30329 USA; Department of Zoology, University of Oxford, Oxford, OX1 3PS UK; Wildlife Conservation Society, Lima, Peru; Tulane School of Public Health and Tropical Hygiene, New Orleans, LA USA; Centers for Disease Control and Prevention, Atlanta, GA 30333 USA

**Keywords:** Retrovirus, Simian foamy virus, Co-evolution, Co-speciation, Nonhuman primates, South America, Peru, Neotropical

## Abstract

**Background:**

Although simian foamy viruses (SFV) are the only exogenous retroviruses to infect New World monkeys (NWMs), little is known about their evolutionary history and epidemiology. Previous reports show distinct SFVs among NWMs but were limited to small numbers of captive or wild monkeys from five (*Cebus*, *Saimiri*, *Ateles*, *Alouatta*, and *Callithrix*) of the 15 NWM genera. Other studies also used only PCR testing or serological assays with limited validation and may have missed infection in some species. We developed and validated new serological and PCR assays to determine the prevalence of SFV in blood specimens from a large number of captive NWMs in the US (n = 274) and in captive and wild-caught NWMs (n = 236) in Peruvian zoos, rescue centers, and illegal trade markets. Phylogenetic and co-speciation reconciliation analyses of new SFV polymerase (*pol*) and host mitochondrial cytochrome *B* sequences, were performed to infer SFV and host co-evolutionary histories.

**Results:**

124/274 (45.2 %) of NWMs captive in the US and 59/157 (37.5 %) of captive and wild-caught NWMs in Peru were SFV WB-positive representing 11 different genera (*Alouatta*, *Aotus*, *Ateles*, *Cacajao*, *Callithrix*, *Cebus*, *Lagothrix*, *Leontopithecus*, *Pithecia*, *Saguinus* and *Saimiri*). Seroprevalences were lower at rescue centers (10/53, 18.9 %) compared to zoos (46/97, 47.4 %) and illegal trade markets (3/7, 8/19, 42.9 %) in Peru. Analyses showed that the trees of NWM hosts and SFVs have remarkably similar topologies at the level of species and sub-populations suggestive of co-speciation. Phylogenetic reconciliation confirmed 12 co-speciation events (p < 0.002) which was further supported by obtaining highly similar divergence dates for SFV and host genera and correlated SFV-host branch times. However, four ancient cross-genus transmission events were also inferred for Pitheciinae to Atelidae, *Cacajao* to ancestral *Callithrix* or *Cebus* monkeys, between *Callithrix* and *Cebus* monkeys, and *Lagothrix* to *Alouatta*.

**Conclusions:**

We demonstrate a broad distribution and stable co-speciation history of SFV in NWMs at the species level. Additional studies are necessary to further explore the epidemiology and natural history of SFV infection of NWMs and to determine the zoonotic potential for persons exposed to infected monkeys in captivity and in the wild.

**Electronic supplementary material:**

The online version of this article (doi:10.1186/s12977-015-0214-0) contains supplementary material, which is available to authorized users.

## Background

Foamy virus (FV), or spumavirus, comprises the only genus of the *Spumavirinae* subfamily of retroviruses [1, 2]. FVs have been reported in several mammalian species, including nonhuman primates (NHPs), cats, cows, horses, and sheep [3–5]. Simian foamy viruses (SFVs) were first described in 1954 as contaminants in primary monkey kidney cultures [6] and since then have been identified in many Old World and New World primate species using a variety of laboratory methods [5, 7]. SFV is the only exogenous retrovirus known to infect New World monkeys. FV is considered non-pathogenic in natural and experimental hosts but systematic, longitudinal studies have not been conducted to verify the apparent non-pathogenicity. Humans can be zoonotically infected with a variety of SFVs originating from Old World monkeys and apes (OWMA) through occupational and natural exposures but demonstrate an apparently asymptomatic though persistent infection [5, 8, 9]. SFV proviral DNA has been shown to be present at low copy numbers in peripheral blood mononuclear cells (PBMCs) and tissues from healthy and immune suppressed animals and infected humans [1, 10–12]. Isolation and/or detection of SFV from the oral mucosa of infected humans and NHPs has also been demonstrated [12–15]. The presence of virus in the oral mucosa and the seroconversion of NHPs at adulthood, a period more prone for biting, supports the hypothesis that transmission occurs via saliva through biting or licking [16, 17]. Moreover, most humans infected with SFV reported NHP bite or scratch exposures with higher prevalences seen in persons with severe bite wounds [10, 18–21].

Phylogenetic analysis has shown species-specific distribution of SFV in OWMA (Catarrhini), indicating a long co-evolution with their natural hosts [22]. However, little is known about the evolutionary history and distribution of SFV in New World primates (Platyrrhini) with the majority of studies done using only animals bred and housed in the US and evidence of infection was only demonstrated using serology [1, 5, 23–25]. Recently, complete SFV genomes have been reported for each of three captive New World monkeys (NWM), including a squirrel monkey (*Saimiri* species), a spider monkey (*Ateles* species), and a common marmoset (*Callithrix jacchus*) [26, 27]. All three NWM SFVs were distinct from those in OWMA and shared less than 50 % amino acid identity in the structural and enzymatic proteins, suggesting that serological assays used to detect SFV from OWMA may be less sensitive for detecting SFV from NWM. While all three NWM SFVs clustered together phylogenetically, a co-evolutionary history could not be verified since only a single sequence from each species is available. In addition, all three were captive animals and cross-species infections from other monkey species could not be excluded.

More recently, with collaborators in Brazil we identified SFV in 18 species of neotropical monkeys from Brazil using PCR-amplification of short (192-bp), highly conserved polymerase (*pol*) sequences, including capuchin (*Cebus* species), owl (*Aotus* sp.), marmoset (*Callithrix* sp.), tamarin (*Saguinus* sp.), squirrel (*Saimiri* sp.), titi (*Callicebus* sp.), saki (*Chiropotes* sp.), and howler (*Alouatta* sp.) monkeys [28]. However, there was not enough phylogenetic information in the highly conserved *pol* sequences in this study to fully resolve the evolutionary histories of all the NWM SFVs from Brazil. Another limitation of the study was the lack of serological testing, which may underestimate the reported prevalence. The authors demonstrated co-evolution of SFV from five NWM species using longer *pol* sequences (520-bp) obtained from *Alouatta* and *Cebus* monkeys in Brazil and *pol* sequences available from complete SFV genomes from spider, squirrel and marmoset monkeys at GenBank. One recent study also demonstrated SFV infection in a small number of three different NWM species captive in the US, including howler, capuchin, and squirrel monkeys [29]. Although these results are informative, the natural history and geographical and species distribution of SFV outside of Brazil and in captive animals elsewhere is thus incomplete.

At least 90 Platyrrhine species live in Central and South America belonging to three families (*Pitheciidae*, *Atelidae*, *and Cebidae*), eight subfamilies [*Callitrichinae* (n = 42), *Cebinae* (n = 14), *Aotinae* (n = 11), *Pitheciinae* (n = 43), *Saimirinae* (n = 10), *Alouattinae* (n = 19), *Callicebinae* (n = 29), and *Atelinae* (n = 24)] [30, 31], and nineteen genera: *Callithr*ix, *Mico*, *Callibella*, *Cebuella*, *Leontopithecus*, *Saguinus*, *Callimico*, *Cebus*, *Saimiri*, *Aotus*, *Callicebus*, *Pithecia*, *Chiropotes*, *Cacajao*, *Alouatta*, *Ateles*, *Brachyteles*, *Lagothrix*, and *Oreonax* [30, 31]. Peru is considered a mega diverse country; with more than 500 species of mammals, 39 of which are primates [32]. Thus, a rich retroviral diversity in Neotropical primates would be expected in Peru like that observed in Brazil, and in OWMAs in Africa and Asia [11, 28, 33]. To better understand the prevalence, geographic distribution, genetic diversity, and evolutionary history of SFV in neotropical primates we tested convenience serum and dried blood spots from primates kept at zoos, rescue centers and illegal trade markets in Peru and in NWMs kept in US zoological gardens and research institutions. Evidence of SFV infection was determined using a combination of serologic and PCR assays followed by sequence analysis to infer phylogenetic and co-evolutionary relationships.

## Methods

### Study populations and sample preparation

**Fig. 1:**
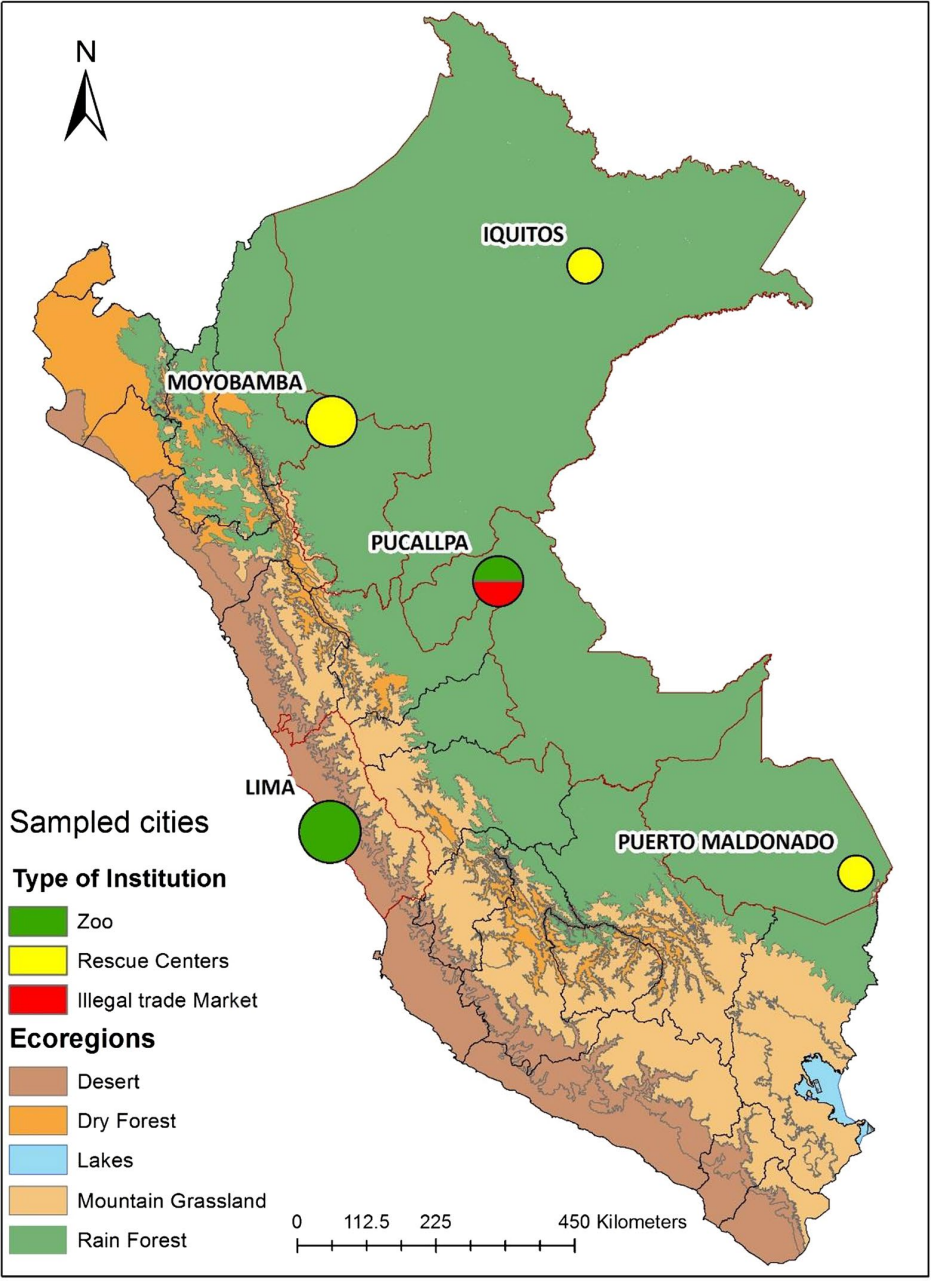
Primate location at zoos, rescue centers and one illegal trade market in Peru. Three zoos participating in the study are located in Lima and one is located in the rainforest region of Pucallpa; one primate rescue center is located near Puerto Maldonado in the southern rainforest, one in Iquitos and two near Moyobamba in the northern lower and upper rainforests. The illegal trade market was located in the rain forest region of Pucallpa

Primates housed at four zoos, four rescue centers and one illegal trade market in five areas of Peru were sampled as part of another study to examine microbial infection in these animals (Fig. 1). Three of the zoos are located in Lima and one is located in the rainforest region of Pucallpa; the primate rescue centers are located near Puerto Maldonado (n = 1), in Iquitos (n = 1) and near Moyobamba (n = 2) in the southern and northern lower and upper rainforests, respectively. The illegal trade market was also located in Pucallpa. All animal work in Peru was approved by the Institutional Animal Care and Use Committee (IACUC) of the US Naval Medical Research Unit No. 6 (NAMRU 6) and approved by the Peruvian Ministry of Agriculture. Specimens were also collected from NWMs at seven US zoos and five research institutions following IACUC approval at each respective institution. Convenience blood samples were collected opportunistically during regular health exams from 211 Neotropical primates in Peru and from 274 captive NWM in the US (Tables 1, 2, 3). Whole blood was collected using standard procedures from the femoral vein with a vacutainer tube without additives. Approximately 125 μl of blood droplets were placed on a dry blood spot (DBS) card or on Whatman FTA filter paper. The remaining blood sample was allowed to clot and the serum was aliquoted and stored at −80 °C. EDTA-treated blood specimens obtained from captive NWMs in the US were processed for plasma and peripheral blood mononuclear cells (PBMCs) as previously described [34]. Plasma samples archived at −80 °C from persons infected with human T-lymphotropic virus (HTLV, n = 65), human immunodeficiency virus (HIV, n = 118), and HIV/HTLV-negative US blood donors (n = 237) that previously all tested negative for OWMA SFV were available for assay development.

**Table 1:** High sensitivity and specificity of new western blot (WB) and PCR assays for the detection of simian foamy virus (SFV) in New World monkeys

Family	Scientific name	Common name	n	WB Pos/PCR Pos	WB Neg/PCR Neg	WB Pos/PCR Neg	WB Neg/PCR Pos
*Atelidae*	*Alouatta palliata*	Mantled howler	1	1	–^a^	–	–
	*Alouatta seniculus sara*	Red howler	3	3	–	–	–
	*Ateles species*	Spider monkey	1	1	–	–	–
	*Ateles belzebuth hybridus*	Colombian brown spider	1	1	–	–	–
	*Ateles fusciceps robustus*	Brown-headed spider	5	3	2	–	–
	*Ateles geoffroyi*	Black-handed spider	17	15	2	–	–
*Cebidae*	*Aotus trivirgatus*	Northern gray-necked owl	10	0	10	–	–
	*Callithrix jacchus*	Common marmoset	10	1	9	–	–
	*Cebus albifrons*	White-fronted capuchin	3	3	–	–	–
	*Cebus apella*	Brown capuchin	28	24	2	2	–
	*Saguinus oedipus*	Cotton-top tamarin	10	0	10	–	–
	*Saimiri species*	Squirrel monkey	1	0	1	–	–
	*Saimiri boliviensis boliviensis*	Bolivian squirrel	6	3	3	–	–
	*Saimiri boliviensis peruviensis*	Peruvian squirrel	7	3	4	–	–
	*Saimiri sciureus*	Common squirrel monkey	1	–	–	–	1
*Pitheciidae*	*Cacajao rubicundus*	Red uakari	2	2	–	–	–
	*Pithecia pithecia*	White-faced saki	1	1	–	–	–
Total			107	61 (57 %)	43 (40.2 %)	2 (1.9 %)	1 (0.9 %)

PCR testing using diagnostic primers to detect 141-bp polymerase sequences in genomic DNA specimens

^a^Dashes indicate an absence of specimen(s) with results in this category

**Table 2:** High sensitivity and specificity of a new EIA for the detection of simian foamy virus (SFV) antibodies in New World monkeys in the US

Family	Scientific name	Common name	n	WB		EIA			
				Pos (%)	Neg (%)	TP (%)	FN (%)	TN (%)	FP
*Atelidae*	*Ateles belzebuth hybridus*	Columbian brown spider	2	2 (100)	–^a^	2	–	–	–
	*Ateles fusciceps robustus*	Brown-headed spider	12	9 (75)	3 (25)	8	1	3	–
	*Ateles geoffroyi frontatus*	Black-handed spider	19	13 (68.4)	6 (31.6)	13	–	6	–
	*Ateles geoffroyi vellerosus*	Mexican spider	14	9 (64.3)	5 (35.7)	9	–	5	–
	*Ateles paniscus chamek*	Peruvian black spider	9	7 (77.8)	2 (22.2)	7	–	2	–
	*Ateles species*	Spider monkey	20	14 (70)	6 (30)	14	–	6	–
	*Alouatta caraya*	Black howler	5	5 (100)	–	5	–	–	–
	*Alouatta palliata*	Mantled howler	2	1 (50)	1 (50)	1	–	1	–
	*Alouatta seniculus sara*	Red howler	2	2 (100)	–	2	–	–	–
	*Alouatta seniculus straminea*	Golden howler	2	1 (50)	1 (50)	1	–	1	–
*Cebidae*	*Aotus trivirgatus*	Northern gray-necked owl	11	1 (9.1)	10 (90.9)	1	–	10	–
	*Callithrix geoffroyi*	Geoffrey’s marmoset	4	2 (50)	2 (50)	2	–	2	–
	*Callimico goeldii*	Goeldii’s marmoset	31	–	31 (100)	–	–	31	–
	*Cebus albifrons*	White-fronted capuchin	3	3 (100)	–	2	1	–	–
	*Cebus apella*	Tufted capuchin	40	37 (92.5)	3 (7.5)	37	–	3	–
	*Leontopithecus rosalia*	Golden lion tamarin	2	2 (100)	–	1	1	–	–
	*Saguinus bicolor*	Pied tamarin	1	–	1 (100)	–	–	1	–
	*Saguinus imperator*	Emperor tamarin	1	–	1 (100)	–	–	1	–
	*Saguinus labiatus*	Red-bellied tamarin	1	–	1 (100)	–	–	1	–
	*Saguinus midas*	Golden-handed tamarin	2	–	2 (100)	–	–	2	–
	*Saguinus mystax*	Moustached tamarin	30	–	30 (100)	–	–	30	–
	*Saguinus oedipus*	Cotton-top tamarin	17	1 (5.9)	16 (94.1)	1	–	16	–
	*Saimiri boliviensis boliviensis*	Bolivian squirrel monkey	7	4 (57)	3 (43)	1	3	3	–
	*Saimiri boliviensis peruviensis*	Peruvian squirrel monkey	7	–	7 (100)	–	–	7	–
	*Saimiri sciureus*	Common squirrel monkey	11	1 (9.1)	10 (90.9)	–	1	10	–
*Pitheciidae*	*Cacajao rubicundus*	Red uakari	2	2 (100)	–	2	–	–	–
	*Callicebus moloch*	Dusky titi monkey	5	–	5 (100)	–	–	5	–
	*Pithecia pithecia*	White-faced saki	12	8 (66.7)	4 (33.3)	7	1	4	–
Total			274	124 (45.2)	150 (54.8)	116 (93.6)	8 (6.5 %)	150 (100 %)	–

*WB* western blot testing, *TP* true positive, *FN* false negative, *TN* true negative, *FP* false positive

^a^Dashes indicate an absence of specimen(s) with results in this category

**Table 3:** Distribution of simian foamy virus in captive and wild caught neotropical monkeys from Peru

Origin	Family	Scientific name	Common name	n	Serology		PCR	
					EIA (%)	WB (%)	141-bp pol (%)	495-bp pol (%)
Illegal trade market	*Atelidae*	*Ateles paniscus chamek*	Peruvian spider monkey	1	0/1	ND	0/1	0/1
		*Lagothrix lagotricha*	Common wooly monkey	2	0/2	ND	0/1	0/1
	*Cebidae*	*Aotus species*	Owl monkey	1	1/1 (100)	1/1 (100)	0/1	0/1
		*Cebus albifrons*	White-fronted capuchin	2	1/2 (50)	1/1 (100)	0/2	0/2
		*Saguinus fusicollis*	Brown mantled tamarin	1	–^a^	–	0/1	0/1
		*Saguinus species*	Tamarin	1	–	–	0/1	0/1
		*Saimiri sciureus*	Common squirrel monkey	4	–	–	1/4 (25)	1/4 (25)
	*Pitheciidae*	*Pithecia monachus*	Geoffrey’s Monk saki	1	1/1 (100)	1/1 (100)	0/1	0/1
Subtotals				13	3/7 (42.6)	3/3 (100)	1/12 (8.3)	1/12 (8.3)
Rescue centers	*Atelidae*	*Alouatta seniculus*	Howler monkey	2	–	–	0/2	ND
		*Ateles bezelbuth*	Long-haired spider monkey	1	0/1	ND^b^	ND	ND
		*Ateles paniscus chamek*	Peruvian spider monkey	28	2/14 (14.3)	2/2 (100)	1/16 (6.3)	3/3 (100)
		*Lagothrix lagotricha*	Common wooly monkey	39	8/36 (22.2)	8/8 (100)	1/5 (20)	2/2 (100)
	*Cebidae*	*Cebus albifrons*	White-fronted capuchin	2	–	–	0/2	ND
		*Cebus apella*	Tufted capuchin	4	1/2 (50)	0/1	0/3	0/1
		*Saimiri boliviensis peruviensis*	Peruvian squirrel monkey	2	–	–	1/2 (50)	1/1 (100)
Subtotals				78	11/53 (20.7)	10/11 (90.9)	3/30 (10)	6/7 (85.7)
Zoos	*Atelidae*	*Alouatta seniculus*	Howler monkey	3	3/3 (100)	3/3 (100)	1/3 (33.3)	1/3 (33.3)
		*Ateles belzebuth*	Long-haired spider monkey	3	0/3	ND	ND	ND
		*Ateles paniscus chamek*	Peruvian spider monkey	7	5/6 (83.3)	4/4 (100)	1/2 (50)	0/1
		*Ateles paniscus*	Red-faced spider monkey	1	0/1	ND	ND	ND
		*Ateles species*	Spider monkey	2	0/2	ND	ND	ND
		*Lagothrix cana*	Peruvian wooly monkey	2	2/2 (100)	2/2 (100)	0/2	ND
		*Lagothrix lagotricha*	Common wooly monkey	14	6/14 (42.8)	5/6 (83.3)	3/11 (27.3)	2/11 (18.2)
	*Cebidae*	*Aotus nancymae*	Peruvian red-necked owl monkey	1	0/1	0/1	ND	ND
		*Aotus nigriceps*	Peruvian night owl monkey	2	0/2	0/2	ND	ND
		*Aotus species*	Owl monkey	3	0/3	ND	ND	ND
		*Callithrix pygmea*	Pygmy marmoset	1	1/1 (100)	0/1	–	–
		*Cebus albifrons*	White-fronted capuchin	7	3/7 (42.8)	2/4 (50)	0/3	ND
		*Cebus apella*	Tufted capuchin	40	29/32 (90.6)	29/29 (100)	11/36 (30.6)	4/13 (30.8)
		*Saguinus fusicollis*	Brown mantled tamarin	15	0/3	ND	0/12	ND
		*Saguinus labiatus*	Red-bellied tamarin	1	1/1 (100)	1/1 (100)	–	–
		*Saimiri boliviensis peruviensis*	Peruvian squirrel monkey	4	0/4	ND	ND	ND
		*Saimiri sciureus*	Common squirrel monkey	6	0/6	0/6	ND	ND
		*Saimiri species*	Squirrel monkey	1	1/1 (100)	0/1	–	–
	*Pitheciidae*	*Callicebus discolor*	Red titi monkey	1	0/1	0/1	–	–
		*Callicebus oenanthe*	Rio Mayo titi monkey	1	–	–	0/1	ND
		*Callicebus species*	Titi monkey	1	0/1	0/1	–	–
		*Pithecia monachus*	Geoffrey’s Monk saki	3	0/3	ND	ND	ND
Subtotals				119	51/97 (52.5)	46/62 (74.2)	16/65 (24.6)	7/28 (25)
Totals				210	65/157 (41.4)	59/76 (77.6)	20/107 (18.7)	14/55 (25.5)

All monkeys at the wet markets and rescue centers were wild caught

*WB* western blot testing

^a^Dashes indicate either serum or FTA specimens were not available for testing

^b^ND, testing not done based on EIA results and/or availability of certain specimen types, i.e. WB testing not done on EIA-negative and/or if FTA cards were not collected for nucleic acid preparation

### Tissue culture isolation and propagation of SFV

PBMCs from a captive spider monkey (*Ateles* species, asp) were stimulated with 10 % IL2 for 48 h and co-cultured with canine thymocytes (Cf2Th) cells using methods reported in detail elsewhere until cytopathic effect (CPE) was observed [34]. Tissue culture supernatant was filtered using a 0.45 uM filter and passaged on fresh Cf2Th cells to propagate SFVasp. SFV from a common marmoset (*Callithrix jacchus*, *SFVcja*) was obtained from the American Type Culture Collection (ATCC VR-919) and propagated in Cf2Th cells [18]. Infection of Cf2Th cells was confirmed by DNA PCR using primers and conditions described below. Infected and uninfected Cf2Th cells were harvested and crude protein lysates were prepared, quantified and used in serological assays as before [34].

### Serological assay development, validation, and specimen testing

Given the high genetic diversity between NWM and OWMA SFVs we developed a new Western blot (WB) assay to detect antibodies to NWM SFV with procedures used successfully to detect a broad diversity of OWMA SFV [21]. Thus, for the WB assay we used antigens from two genetically diverse NWM SFVs, SFVasp and SFVcja, that share about 65 % genetic identity [26] to allow broad serologic detection of SFV in all three Platyrrhine families. Protein concentrations of the lysates were determined using the BioRad DC Protein Assay (Hercules, CA, USA). Plasma or serum samples were diluted 1:50 and reacted separately to 150 μg of infected and uninfected cell lysates overnight at 4 °C after protein separation through 4–12 % polyacrylamide gels and transfer to Nytran membranes, as previously described [34]. Seroreactivity was detected using peroxidase-conjugated protein A/G (Pierce, Rockford, IL, USA) and chemiluminescence (Amersham, Uppsala, Sweden) [34]. Seroreactivity to both Gag p68 and p72 precursor proteins with an absence of similar reactivity to antigen from uninfected Cf2Th cells was interpreted as seropositive. Specimens without reactivity to either Gag protein were considered seronegative.

The SFV WB assay was validated with serum and plasma samples from 15 different species of NWMs (n = 107, Table 1). The infection status of these primates was determined by PCR analysis using newly developed generic *pol* primers as described below. Specificity of the WB assay was also determined using sera from 118 persons infected with HIV-1/2, 65 persons with HTLV-1/2 infection and 237 HIV/HTLV-negative sera from US blood donors. Cross-reactivity of selected sera from different SFV-infected NWM genera to SFV antigens derived from Old World monkeys and apes (SFVagm, African green monkey) and SFVcpz (chimpanzee) was also performed by WB testing to further evaluate assay specificity.

We also developed a new enzyme immunoassay (EIA) to facilitate rapid screening of a large number of specimens for antibodies to NWM SFV. Serum or plasma samples were diluted 1:100 in assay diluent and tested in duplicate in two different microtiter wells coated with crude cell lysates from Cf2Th cells infected with SFVasp and SFVcja in a single well and uninfected Cf2Th lysates in a separate well to assess assay specificity. Each specimen was tested in duplicate. Replicate sample optical density (OD) values were averaged and adjusted ODs of reactivity to SFV minus that to the uninfected antigens were calculated as described before [35].

### SFV and host PCR

Genomic DNA was extracted from FTA/DBS cards using the QIAamp DNA Mini Kit (QIAGEN) as described by the manufacturer. DNA lysates were prepared from PBMCs from monkeys captive in the US and the integrity of the extracted DNA was validated using ß-actin PCR as described in detail elsewhere [18]. All DNA samples were first screened for SFV sequences using a novel semi-nested PCR that utilizes generic *pol* primers. These primers were designed using an alignment of sequences from the three complete SFV genomes available at GenBank from marmoset (SFVmar), squirrel (SFVsqu), and spider monkey (SFVspm) (accession numbers GU356395, GU356394, and EU010385, respectively) [26, 27].

For the first PCR, 0.5 μg of DNA was applied to 50 μl of reaction mixture containing 1× buffer with 1.5 mM MgCl_2_, 800 uM dNTPs, 2 ng/μl of primary primers (SIF5N 5′ TAC ATG GTT ATA CCC CAC KAA GGC TCC TCC 3′ and SIR5N 5′ AAT AAW GGA TAC CAC TTT GTA GGT CTT CC 3′) and 1.25 U AmpliTaq DNA polymerase (Applied Biosystems) with the following conditions to generate a 282-bp sequence: five cycles at 94 °C for 1 min; 37 °C for 1 min and 72 °C for 1 min, then 35 cycles at 94 °C for 1 min; 50 °C for 1 min and 72 °C for 1 min, with a final extension at 72 °C for 1 min. For the second PCR, 2.5 μl of primary product was added to 50 μl nested PCR mixture containing the same concentration of components as the first PCR except using the nested primers SIP4N (5′ TGC ATT CCG ATC AAG GAT CAG CAT T 3′ and SIR1NN (5′ GTT TTA TYT CCY TGT TTT TCC TYT CCA CCA T 3′) to generate a 141-bp *pol* sequence. Nested PCR conditions were 40 cycles at 94 °C for 1 min; 50 °C for 1 min and 72 °C for 1 min. 20 μl of nested PCR product of each sample was loaded onto a 1.8 % agarose gel for electrophoresis analysis. Samples positive using the generic *pol* primers were subjected to additional PCR testing to obtain longer fragments containing adequate sequence information for resolution by phylogenetic analysis. Primary (SNF3 5′ GAT AAR TTG GCW RYM CAA GGW AGT TAT 3′ and SNR3 5′ GAR GTR AAT GCT GAT CCT TGA TCG GAA T 3′) and semi-nested PCR primers (SNF3 and SNR4 5′ GAA GGA GCY TTH GTG GGG TAT AAC CA 3′) were used to amplify 581 and 495-bp *pol* sequences, respectively, using 35 cycles of standard PCR and an annealing temperature of 50 °C.

Primate host species taxonomic classification was determined by analysis of 975-bp cytochrome *B* (*CytB*) mitochondrial DNA (mtDNA) sequences obtained by one-step PCR using primers L14724 (5′ CGA AGC TTG ATA TGA AAA ACC ATC GTT G 3′) and Mus15398 (5′ GAA TAT CAG CTT TGG GTG TTG RTG 3′) as previously described [28].

### Statistical analysis

MedCalc v12.5.0 was used to perform receiver operator curve analysis of EIA data and infer assay cutoff values and for 2 × 2 tables for determining assay sensitivity, specificity, positive predictive value (PPV), negative predictive value (NPV), and accuracy. Online statistical tools available at VassarStats (http://vassarstats.net/) were used to determine associations using Chi square or Fisher exact probability tests. When known, NWMs were stratified by age with adults being >4 years old, sub-adults 3–4 years old, juveniles 1 <3 years old, and infants <1 year old.

### Sequence analyses

Amplified products were purified, quantified, and sequenced on both strands using the Big Dye v.3.1 sequencing kit (Life Technologies, Carlsbad, USA) and an automated ABI 3130XL Genetic Analyzer and edited with SeqMan v7.0 (DNASTAR, Madison, USA). New SFV and *CytB* sequences were aligned with those available from NWM retrieved from GenBank by using the Clustal W program implemented in MEGA v6 [36]. Quality of the alignments was verified using the program GUIDANCE (http://guidance.tau.ac.il/). Percent nucleotide identities were determined using the program Geneious Pro v6.1.3.

### Recombination detection

The absence of genetic recombination in the *pol* alignments was confirmed using Bootscan, Geneconv, MaxChi, Chimera, and RDP within the program RDP v3 using the parameter defaults [37].

### Phylogenetic reconciliation analysis

A Bayesian phylogeny of 74 NWM SFVs was estimated from a manually-curated *pol* alignment (412 nt) by using MrBayes 3.2.1 [38] without imposing a molecular clock which may influence and/or bias the tree topology estimation. Six OWMA SFV sequences (PFV; Y07725, SFVcpz; U04327, SFVgor; HM245790, SFVora; AJ544579, SFVmac; NC_010819, and SFVagm; M784895) were included as an outgroup. Nine sequences in the alignment were 3351 nt long (PFV, SFVcpz, SFVgor, SFVora, SFVmac, SFVagm, SFVmar; GU356395, SFVspm; EU010385, and SFVsqu; GU356394) and were included as ‘backbones’ to increase the power of deep node separation. The GTR+I+Γ(4) nucleotide substitution model was used. Two independent Markov chain Monte Carlo (MCMC) chains were run for 50 million steps with the initial 25 % discarded as burn-in. Trees and parameters were logged every 2500 steps thereafter. A metropolis coupling algorithm was applied to improve the MCMC samplings, using the setting of 3 hot and 1 cold chains. A Bayesian phylogeny of 156 NWM hosts was also estimated by using the same protocol from a manually-curated *CytB* alignment (618 nt). However, unlike the SFV phylogeny, the host phylogeny was rooted according to the tree in Perelman et al. [30]. All alignments are available from the authors upon request. The convergence of estimated parameter values was diagnosed using potential scale reduction factors (PSRFs). PSRFs of all parameters were ~1.000, indicating that they were all well sampled from their posterior distributions and had converged.

We then compared the topologies of the consensus NWM SFV and host phylogenies to infer potential co-speciation events by using the co-phylogeny reconstruction software Jane v4 with the following settings: generation number = 50 and population size = 100 [39]. In total, four reconciliation analyses were performed: (1) at the genus level, (2) species level—conservative tree collapsing, (3) species level—overall, and (4) all sequences (see “Results and discussion” for details). In the last analysis, NWM SFVs for which the corresponding host sequences were not available were excluded from the analysis. The vertex-based cost mode was used with costs set to maximize the number of co-speciation events (co-speciation = −1, duplication = 0, duplication and host switch = 0, loss = 0, and failure to diverge = 0). To assess the probability of observing the inferred co-divergence number by chance, a null distribution was calculated by using the random tip mapping method implemented in Jane v4 with the settings of generation number = 50, population size = 100, and sample size = 500 [39].

### Evaluation and refining the co-speciation model

To evaluate the inferred co-speciation events and refine the co-evolution model, we first inferred the dates for some of the co-speciation events that could be mapped conclusively onto the tree, directly from the host dates estimated in [30]. Four dates were inferred in total, including (1) the NWM-OWMA FV separation date, (2) the branching date of the *Cebus xanthosternos* SFV lineage, (3) the branching date of the *Ateles chamek* SFV lineage, and (4) the separation date between the *Alouatta belzebul* and *Alouatta sara* SFV lineages. These dates were then used to estimate the divergence dates of other nodes in the SFV tree, under the Bayesian phylogenetic framework using BEAST 1.8.2 [30, 40]. The BEAST analysis used the SRD06 nucleotide substitution model, the Yule speciation process, a relaxed log-normal molecular clock and the default prior settings. The tree topology was fixed to the one we obtained from the MrBayes analyses. The Bayesian MCMC was run for 50 million steps with the initial 25 % discarded as burn-in. Trees and parameters were logged every 2500 steps thereafter. Parameter value convergence and sampling independency were manually inspected using the program Tracer included in the BEAST package. Effective sample sizes of all parameters were >200, indicating that they were all well sampled and had converged. We then compared the estimated SFV evolutionary timescales to those of their hosts and refined our SFV-host co-evolutionary model. The tree with the maximum product of the posterior clade probabilities (maximum clade credibility tree) was constructed from the posterior distribution of the sampled trees with the program TreeAnnotator v.1.8.2. Node heights were calculated from the posterior distribution of the trees and viewed in FigTree v.1.3.1.

To further evaluate the co-evolutionary model, an SFV-host divergence correlation analysis was performed by manually identifying a subset of SFV-host co-diverging branches based on the refined model, and examining if the SFV and host divergences are linearly correlated. The linear correlation and its *p* value were calculated using the *lm* function implemented in R (https://cran.r-project.org). The coefficient of determination (R^2^) and the *p* values were also derived.

### GenBank accession numbers

All new *CytB* and SFV sequences generated during our study have been deposited at GenBank with the accession numbers KR902362-KR902495.

## Results and discussion

### SFV NWM PCR, WB and EIA validation

To determine the sensitivity of the diagnostic NWM SFV PCR assay both SFVasp and SFVcja tissue culture DNA lysates were diluted 10 fold with lysis buffer from 10^−1^ to 10^−8^ representing 0.1 μg to 0.01 pg cellular DNA and PCR tested. The diagnostic primers were able to detect both SFVasp and SFVcja sequences between 0.1 and 1.0 pg (10^−6^ to 10^−7^ dilutions). DNA lysates prepared from PBMCs collected from a human without exposure to NWMs were consistently negative in all assay runs. We also determined the copy number sensitivity of the diagnostic primers using SFVasp and SFVcja *pol* plasmid clones to be 10 and 1 copies each, respectively, which is similar to that reported recently using an SFVsqu *pol* plasmid [29]. To evaluate the ability of these primers to detect a broad range of SFV in NWMs we screened PBMC lysates from 107 captive NWMs representing nine genera and 17 species (Table 1). SFV sequences were detected in 61 individual animals (57 %) from seven genera in each of the three NWM families *Pitheciidae*, *Atelidae*, and *Cebidae*, including *Cebus apella* (24/28, 85.7 %), *Cebus albifrons* (3/3, 100 %), *Alouatta palliata* (1/1), *Alouatta seniculus sara* (3/3, 100 %), *Callithrix jacchus* (1/10, 10 %), *Ateles* species (1/1), *Ateles belzebuth hybridus* (1/1, 100 %), *Ateles geoffroyi* (15/17, 88.2 %), *Ateles fusciceps robustus* (3/5, 60 %), *Saimiri boliviensis* (3/6, 50 %), *Saimiri boliviensis peruviensis* (3/7, 42.9 %), *Cacajao rubicundus* (2/2), and *Pithecia pithecia* (1/1). These results demonstrate the ability of the diagnostic primers to broadly detect SFV diversity in a wide range of divergent NWM species.

**Fig. 2:**
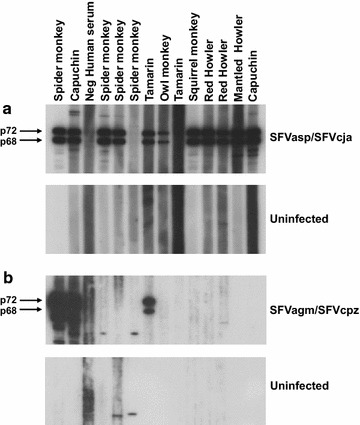
Detection of plasma/serum antibodies to SFV from spider monkey (SFVasp) and marmoset monkey (SFVcja) using a combined antigen western blot (WB) assay. **a** *Upper panel* shows seroreactivity of representative neotropical primate samples to the combined NWM SFV antigens from spider monkey (*asp* *Atele*s species) and marmoset (*cja* *Callithrix jacchus*) tissue cultures; *lower panel* shows reactivity to crude cell lysate antigens from uninfected canine thymocytes (Cf2Th). **b** *Upper panel* shows seroreactivity of representative Neotropical primate samples to combined Old World monkey (*AGM* African green monkey) and ape (*cpz* chimpanzee) SFV antigens except *lanes* *1* and *2* are plasma samples from SFVagm- and SFVcpz-infected humans as positive controls; *lower panel* shows reactivity to crude cell lysate antigens from uninfected Cf2Th. Seroreactivity was defined as those specimens with reactivity specific to the diagnostic Gag doublet proteins in the combined viral antigens. *Lane* *3* is a pedigreed negative human plasma control

Next, plasma from all 107 NWMs was tested using the new WB assay for comparison with the PCR results to infer the sensitivity and specificity of each assay. Representative WB results for a variety of NWM species are shown in Fig. 2. Very little cross-reactivity of NWM sera to OWMA SFV antigens was observed, supporting the specificity of the NWM SFV WB test (Fig. 2b). Concordant WB and PCR results were obtained for 104 specimens, giving a 97.2 % accuracy of each assay for SFV detection (Table 1). Two specimens from *Cebus apella* were WB-positive but PCR-negative and conversely one sample from a *Saimiri sciureus* was WB-negative but PCR-positive. Repeat WB testing of this animal using a serum sample collected 3 years later gave concordant results (data not shown). Thus, the WB assay was found to have a sensitivity, specificity, PPV, NPV, and accuracy of 96.8, 99.8, 98.4, 99.6, and 99.6 %, respectively. The sensitivity, specificity, PPV, NPV, and accuracy of the diagnostic PCR test was 98.4, 95.6, 96.8, 97.7, and 97.2 %, respectively.

To efficiently screen large numbers of specimens for evidence of NWM SFV infection we also developed a microtiter-based EIA. For this purpose, we expanded screening of serum and plasma specimens available from an additional 167 NWMs that were also tested by WB analysis for a total of 274 specimens (Table 2). PBMC DNA was not available from the majority of these 167 animals for PCR testing. Of these, 124 (45.2 %; 39.3–51.4 % 95 % CI) were WB-positive and 150 (54.8 %) were WB-negative (Table 2). EIA specificity was also determined using a total of 417 specimens from HIV-1-infected persons (n = 56), HIV-1/2 infections (n = 59), HTLV-1/2 infection (n = 65), and US blood donors (n = 237) that all tested negative in the WB assay (Table 3).

An adjusted OD ≥0.235 was set as a cutoff value for seroreactive samples using receiver operator curves (ROC) generated in the MedCalc software program based on assay validation with the WB-confirmed specimens (Tables 1, 2). Using this cutoff, the EIA assay sensitivity, specificity, PPV, PNV, and accuracy was 93.6, 97.7, 91.0, 98.4, and 96.9 %, respectively. The EIA gave concordant positive and negative results for all NWM specimens except for false negative results for eight WB-positive samples (6.5 %) from four squirrel, one capuchin, one saki, one spider, and one tamarin monkey (Table 2). All eight specimens showed weak seroreactivity in the WB test. False-positive results were obtained with 13 WB-negative human specimens (3.1 %), including seven blood donors, three HTLV-positive, and three HIV-positive samples (Table 4).

**Table 4:** High specificity of a new EIA for the detection of simian foamy virus (SFV) antibodies in humans

Population	n	WB		EIA			
		Pos (%)	Neg (%)	TP	FN	TN (%)	FP (%)
HIV-1-infected	56	–^b^	56 (100)	–	–	55 (98.2)	1 (1.8)
HIV-1/2-infected	59	–	59 (100)	–	–	57 (96.6)	2 (3.4)
HTLV-1/2-infected	65	–	65 (100)	–	–	62 (95.4)	3 (4.6)
US blood donors^a^	237	–	237 (100)	–	–	230 (97.1)	7 (2.9)
Total	417	0	417 (100)	–	–	404 (96.9)	13 (3.1)

*WB* western blot testing, *TP* true positive, *FN* false negative, *TN* true negative, *FP* false positive

^a^Blood donors previously tested negative for antibodies to SFV from Old World monkey and apes

^b^Dashes indicate an absence of specimen(s) with results in this category

### SFV prevalence and distribution in captive NWM monkeys in the US and in captive and wild monkeys from Peru

The overall SFV prevalence in the 274 captive seroreactive monkeys from the US was 45.2 % (39.3–41.5 % 95 % CI) and ranged from 0 to 100 %, but included species with small numbers of representatives such as Emperor tamarins (0/1, 0 %) and brown spider monkeys (2/2, 100 %) (Table 2). For those species with at least 10 animals, SFV prevalence was greatest in tufted capuchins (37/40, 92.5 %; 78.5–98 % 95 % CI) followed by brown-headed spider monkeys (9/12, 75 %; 42.8–93.3 % 95 % CI), spider monkeys (14/20, 70 %; 45.7–87.2 % 95 % CI), black-handed spider monkeys (13/19, 68.4 %; 43.5–86.4 % 95 % CI), white-faced sakis (8/12, 66.7 %; 35.4–88.7 % 95 % CI), Mexican spider monkeys (9/14, 64.3 %; 35.6–86 % 95 % CI), common squirrel monkeys (1/11, 9.1 %; 0.5–42.9 % 95 % CI), northern grey-necked owl monkeys (1/11, 9.1 %; 0.5–42.9 % 95 % CI), and cotton-topped tamarins (1/17, 5.9 %; 0.3–30.8 % 95 % CI). SFV was absent in mustached tamarins (0/30), whose ages ranged from 2.6 to 10.5 years old, and also in Goeldii’s marmosets (0/31; ages not available).

180 sera and 178 DBS or FTA-prepared blood samples were collected from 18 primate species at zoos (n = 130), rescue centers (n = 78) and illegal trade markets (n = 7) in Peru (Fig. 1; Table 3). All monkeys at the trade markets and rescue centers were wild caught. Overall, 59/157 (37.6 %, 30.1–45.7 % 95 % CI) of these NWMs had antibodies against SFV. For animals with serum or plasma available, a higher SFV seroprevalence was observed at zoos (46/97, 47.4 %; 37.2–57.8 % 95 % CI) and at illegal trade markets (3/7, 42.68.1–64.6 %95 % CI) than at rescue centers (11/53, 18.9 %; 9.9–32.4 % 95 % CI) (Table 3), though this difference was not statistically significant (*p* = 0.337 and 0.228, respectively). However, the higher SFV prevalence at zoos was significant compared to that found at rescue centers (*p* = 0.001). For the three species with more than 10 animals, the highest prevalence was seen in *C. apella* (90.6 %), *L. lagotricha* (42.8 % at zoos and 22.2 % at rescue centers), and *A. chamek* (14.3 %). Our observed wide distribution of SFV in the three NWM families is similar to that reported in Brazil [28]. However, for the first time we identify SFV infection of *L. lagotricha* and *L. cana*, *Ateles chamek* and *A. belzebuth*, *Pithicea monachus*, *Saguinus labiatus*, and *Saimiri boliviensis* which were either not sampled or were under sampled in the Brazilian study (Table 3 and [28]). The overall WB prevalence of SFV in NWM at the zoos in Peru (47.4 %) is comparable to that seen in monkeys at US zoos (45.2 %). However, our SFV prevalence rates are somewhat higher that those reported in Brazil (14–30 %) but which used only PCR testing and also which had numerous species in that study which were under sampled [28]. It is also not clear what impact the absence of ages for some animals in our study may have had on the observed prevalences.

SFV seroprevalence in captive NWM in the US was nearly identical in male (41/73, 56.2 %) and female (58/98, 59.2 %) animals. Prevalence increased with age in both males and females and ranged from zero percent in two infants, 30–50 % in juveniles, 50–58 % in sub-adults, and 55–64 % in adults. Age and sex were not available for all monkeys, including the negative Goeldii’s marmosets. The SFV prevalence in males (19.4 %, 14/72) compared to females (26.1 %, 23/88) in Peru was not statistically significant (*p* = 0.209) with gender available for most animals at zoos (male = 47, female = 36) and rescue centers (male = 25, female = 52) only. These results are similar to the equal distribution reported in the captive NWMs in the US (Table 2), in captive adult NWMs in Brazil, and other studies of SFV-infected OWMA [1, 17, 28, 41]. However, the SFV prevalence in both males and females in US zoos was significantly higher than those combined in zoos and rescue centers in Peru (*p* < 0.0001).

Seroprevalence was higher in adult (17/53, 32.1 %) and sub-adult (1/1, 100 %) animals at rescue centers and zoos in Peru than in juvenile (4/24, 16.7 %) monkeys, but the totals may be too low for a statistically informative comparison. Sex and ages were not recorded for the monkeys captured at the illegal trade market which may limit estimating the overall prevalence in this setting in Peru. Nonetheless, the finding of higher SFV prevalences in adult animals than in juveniles is consistent with that reported for OWMA [1, 17, 41], indicating an increased risk of SFV transmission associated with aggressive behaviors, such as biting and scratching, that occur as monkeys approach sexual maturity.

SFV provirus DNA was detected in 20/107 (18.7 %) FTA samples from Peruvian NWMs by PCR using the highly degenerate *pol* primers (Table 3). The majority of PCR-positive specimens were from animals housed at zoos (16/65, 24.6 %), followed by rescue centers (3/30, 10.0 %). SFV PCR detection was distributed across a wide range of species, including *Cebus apella* (n = 11), *Lagothrix lagotricha* (n = 4), *Ateles chamek* (n = 2), *Alouatta seniculus* (n = 1), *Saimiri boliviensis* (n = 1), and *Saimiri sciureus* (n = 1). One *L. lagotricha* and both PCR-positive *Saimiri* species were from rescue centers. These results are similar to the 24.1 % PCR prevalence reported in mostly captive NWMs from Brazil [28], but are about half that (57.3 %) observed in captive US NWMs in our current study (Table 1).

### Co-evolutionary history of NWM SFVs and their hosts

**Fig. 3:**
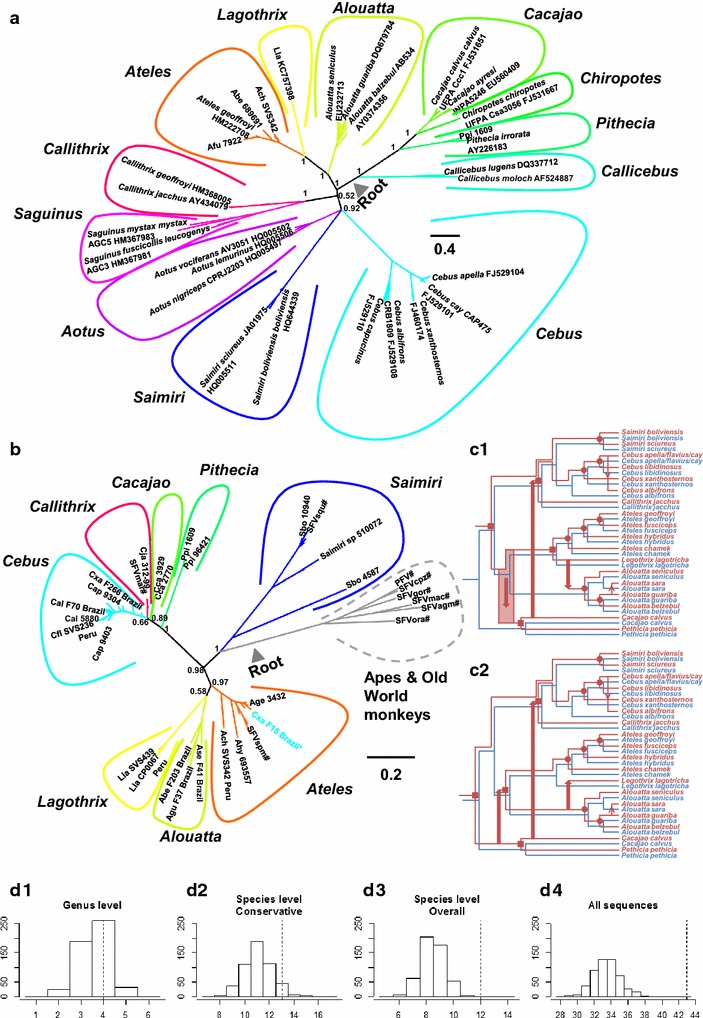
Co-speciation history of New World monkeys (NWM) and simian foamy viruses (SFVs). **a** A consensus Bayesian phylogeny of NWM hosts, estimated from an alignment of cytochrome *B* nucleotide sequences (156 sequences, 618 nt) by using MrBayes 3.2.1 [38]. The tree was rooted according to the phylogeny in [30]. **b** A consensus Bayesian phylogeny of NWM FVs, estimated from an alignment of polymerase nucleotide sequences (74 sequences, 412 nt), and rooted using six ape and Old World monkey SFVs. ‘Backbone’ sequences are indicated with ‘*hash*’. ‘Cxa F15 Brazil’ (written in *blue* and indicated with an *asterisk* ‘*’) was isolated from a *Cebus xanthosternos* monkey in Brazil, but was found to be placed well within the clade of *Ateles* SFVs. The roots of the trees are indicated by *grey triangles*. *Numbers* on nodes are posterior probability node supports. The *scale bars* are in the units of substitutions per site. The comprehensive SFV and host trees are shown in Additional file 1: Figure S1. **c** Competing models for the co-evolutionary histories of NWM SFVs (*red*) and their hosts (*blue*) at the level of species inferred by Jane v4 [39]. The directions of transmissions are indicated by *red arrows*. *Small arrows* indicate cross-species transmissions, and *large arrows* indicate cross-genus transmissions. The *red* *transparent bars* show the uncertainty of the cross-species transmission timing. Four co-speciation events at the genus level are indicated by *solid red squares*, and those at the species level are indicated by *solid red circles*. Note that, the trees are not scaled to time. **d** The distributions of the number of co-speciation events expected to occur by chance, estimated by using random tip mapping method implemented in Jane v4 [39] (sample size = 500); **d1** genus level; **d2** species level, conservative tree collapsing method; **d3** species level, overall; and **d4** all sequences. The *dotted line* indicates the actual observed number of co-speciation events inferred by Jane v4 [39]. See Additional file 2: Table S1 for a complete list of species codes used in the study; PFV is primate foamy virus which is the new name given to HFV (human foamy virus)

To investigate the co-evolutionary history of NWM SFVs and their hosts, we first estimated their phylogenies, and subsequently compared the two topologies to reconstruct possible co-phylogenetic histories. The host tree contained confirmed SFV-positive primate species and randomly selected SFV-negative monkeys for which DNA specimens were available. The tree was reconstructed by using phylogenetic analysis of 618-bp *CytB* sequences from 158 taxa, of which 32 and 44 are from Peru and US zoo monkeys, respectively, and was rooted according to the phylogeny in [30]. Thirteen *CytB* sequences were from Brazilian NWMs reported in a recent paper investigating SFV diversity [28]. We found that *CytB* sequences of the same host genus cluster together forming monophyletic clades, and that the topology of the estimated tree is generally similar to the one obtained by Perelman et al. [30] (Fig. 3a; Additional file 1: Figure S1, right) with one exception; while we estimated *Aotus* to be a sister clade to the *Saimiri*-*Cebus* clade, Perelman et al. found *Aotus* to be a sister clade of the *Callithrix*-*Saguinus* clade. This, however, would not affect our SFV-host co-phylogenetic analysis since our SFV tree does not contain any *Aotus* FVs. Others also have shown that the placement of *Aotus* in NWM phylogeny is not completely resolved [31].

Phylogenetic analysis of the short *pol* SFV sequences (85-bp without primer sequences) was not performed since there is not enough phylogenetic information present in the alignment to accurately resolve the branching orders. Similar results have been reported using slightly longer *pol* sequences (138-bp) that overlaps our diagnostic PCR region [28]. In this study, we successfully amplified longer *pol* sequences (495-bp) from 14 of the 20 (70 %) Peruvian monkeys, including five *L. lagotricha*, five *C. apella*, two *Saimiri* species, one *Ateles chamek*, and one *Alouatta seniculus*. We also obtained 42 additional 495-bp *pol* sequences using blood specimens from NWMs captive in the US, which were used for the PCR and serologic validation assays, and from the SFVcja and SFVspm tissue culture DNAs. SFV *pol* sequences (~383-bp) from three captive NWM species (howler, capuchin, and squirrel) reported by Stenbak et al. [29] only overlap our *pol* sequences by about 197 nucleotides and were from species already in our dataset; thus they were not included in our analyses. The phylogeny of these 58 new *pol* sequences with those available from GenBank, including 13 from Brazilian NWMs [28], is shown in Fig. 3b and Additional file 1: Figure S1 (left).

We noticed that there are some inconsistencies between the SFV tree estimated herein and the one obtained in our previous study that was based on an analysis of short *pol* sequences (17 sequences, 276 nt) [28]. In our previous study, we found ‘Cxa F266’ isolated from a *C. xanthosternos* clustered with a *Callithrix* SFV, suggesting that this virus may represent a recent cross-genus transmission. However, in this work we found Cxa F266 to be more closely related to *Cebus* SFVs than to *Callithrix* SFVs which is consistent with a phylogenetic analysis of long terminal repeat-*gag* sequences (18 sequences, 265 nt) that was also conducted in our previous study [28]. Combined, it is therefore more likely that Cxa F266 is in fact a *Cebus* SFV, and hence does not represent a cross-genus transmission. Another inconsistency found is that while our present study showed that SFV from a spider monkey (SFVspm) is more closely related to SFV from a common marmoset (SFVmar) than to SFV from a squirrel monkey (SFVsqu) (which is also consistent with previous Gag and Pol protein analyses [26, 42]), our previous work [28] showed that SFVspm is more closely related to SFVsqu than SFVmar. These two disparities are likely because the *pol* sequences used in our previous study were too short and/or the number of SFV sequences used in the analysis was too low.

Overall, phylogenetic analyses show that SFVs isolated from the same host genus tend to cluster together forming monophyletic clades. SFV ‘Cxa F15 Brazil’ is the only exception to this; as shown in a previous study [28], this virus was isolated from a *Cebus xanthosternos* monkey but instead clustered within the *Ateles* SFV clade. This likely represents a recent cross-genus SFV transmission which has been reported previously in captive and wild OWMAs [22, 43, 44]. By comparing the topologies of the viral and host trees, another three cross-genus transmissions were inferred: (1) from an ancestral Atelidae monkey to an ancestral Pitheciinae monkey (Fig. 3c1) or vice versa (Fig. 3c2), (2) from an ancestral *Cacajao* monkey to the lineage giving rise to *Cebus* and *Callithrix* monkeys, and (3) from an ancestral *Lagothrix* monkey to an ancestral *Alouatta* monkey (Fig. 3c1) or vice versa (Fig. 3c2). Four potential co-speciation events at the level of viral genera were also inferred: (1) the divergence of the *Pithecia* SFV lineage from *Cacajao* SFV lineage, (2) the separation of the *Ateles* SFV lineage from *Lagothrix* (Fig. 3c1) or *Alouatta* (Fig. 3c2) SFV lineage, (3) the split of an ancestral *Cebus* SFV from an ancestral *Callithrix* SFV, and (4) the divergence of the *Saimiri* SFV lineage from *Atelidae* (Fig. 3c1) or *Pitheciidae* (Fig. 3c2) SFV lineage. Nevertheless, it is noteworthy that the clade of *Lagothrix* and *Alouatta* is weakly supported (posterior probability = 0.58, Fig. 3b). An alternative scenario could imply that *Atelidae* SFVs and their hosts co-diverged with one another, decreasing the number of cross-genus transmissions by one and thereby increasing the number of co-divergence events by one. These four (or five) co-divergence events, however, are not greater than expected to occur by chance [random tip mapping: sample size = 500, p (4 co-speciation events) = 0.516, p (5 co-speciation events) = 0.062, Fig. 3d1].

SFV transmissions among closely related NWMs are also relatively common. *Cebus* FVs are a clear example of this. Phylogenetic analyses show that *C.apella*, *C. flavius*, and *C. cay* FVs, as well as their hosts, cluster together without forming clear phylogenetic structures (Additional file 1: Figure S1), indicative of frequent cross-species transmissions among these closely related monkeys with overlapping habitats. A few transmissions within the clades of *Ateles* monkeys and *Saimiri* monkeys were also observed (Additional file 1: Figure S1). To examine whether or not SFVs stably co-speciate with their NWM hosts at the species level, we collapsed the clades comprising host and viral sequences of the same species into one representative sequence and compared the trees. In total, 13 co-speciation events were inferred. Again, this is not greater than expected to occur by chance (random tip mapping: sample size = 500, p = 0.108, Fig. 3d2). Nonetheless, it is important to note that this method is highly conservative, retaining all transmissions in the trees. Since our samples were collected from a market, zoos, and rescue centers where cross-species transmissions can readily occur due to extreme close proximity, it is possible that this method might be too conservative and the noise from spurious recent cross-species transmissions might overwhelm the signal of the FV-host co-speciation history.

To examine this possibility, we pruned the FV tree further such that it contains only one FV per one host species, of which the phylogenetic placement represents the majority of its kind. We then compared this pruned FV tree to the host species tree. 12 potential co-speciation events were inferred in total (Fig. 3c), which are greater than expected to occur by chance (random tip mapping: sample size = 500, p < 0.002, Fig. 3d3). Combined with the results above, this finding suggests that, overall, NWM SFVs stably and broadly co-diverge with their hosts at the species level over the long timescale; however, cross-species and genus transmissions are not rare. Lastly, we compared the entire SFV tree with the host tree (Additional file 1: Figure S1). In total, 43 potential co-speciation events were inferred, which is greater than expected to occur by chance (Random tip mapping sample size = 500, p < 0.002, Fig. 3d4). These findings suggest that NWM FVs stably co-speciate with their hosts at the level of subpopulation also, and are consistent with the results from a previous study that was based on OWMA FVs [18].

### Evaluating and refining the co-speciation model

**Fig. 4:**
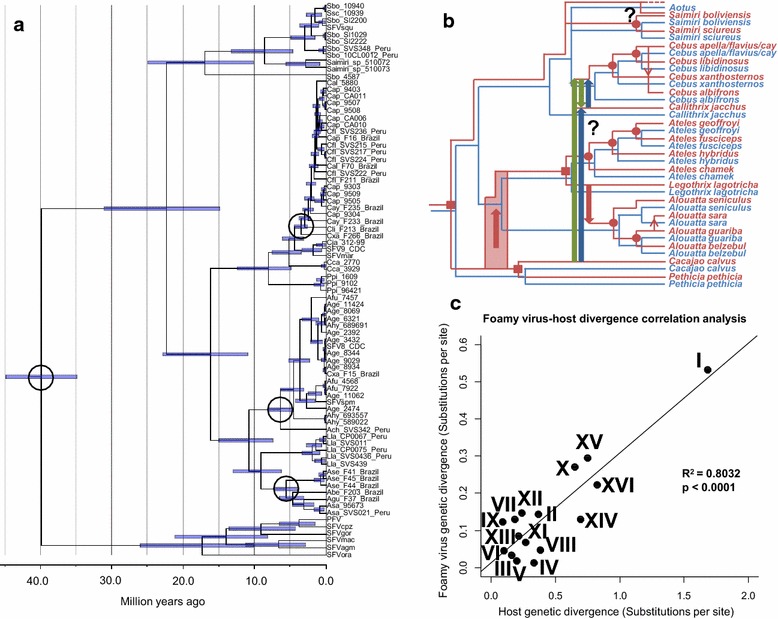
Evaluating and refining simian foamy virus (SFV)-host co-evolutionary model. **a** Calibrated maximum clade credibility Bayesian SFV phylogeny, estimated from an alignment of polymerase nucleotide sequences (74 sequences, 412 nt), using BEAST 1.8.0 [40]. The topology was fixed to the one we obtained from the MrBayes analyses. The calibrating nodes are encircled. The *bars* represent the uncertainty of the estimated node heights. The timescale is in millions of years. See Additional file 2: Table S1 for a complete list of species codes used in the study; PFV is primate foamy virus which is the new name given to HFV (human foamy virus). **b** Refined co-evolutionary history of SFVs (*red*) and their hosts (*blue*). *Red dotted* *branch* represents a possible *Aotus* ghost FV lineage that has not been sampled in our study. The directions of cross transmissions are indicated by *arrows*. *Small arrows* indicate cross-species transmission events, and *large arrows* indicate cross-genus transmission events. The *red transparent bar* shows the uncertainty of the cross-species transmission timing. Two alternative possible SFV cross-species transmission scenarios involving *Cacajao*, *Cebus*, and *Callithrix* monkeys are shown in *green* and *blue*. ‘*Question mark*s’ indicate ambiguous cross-species transmission pathways. Co-speciation events at the genus level are indicated by *solid red squares*, and those at the species level are indicated by *solid red circles*. The trees are not scaled to time. **c** SFV-host divergence correlation analysis. *Black dots* represent co-diverging branches identified under the refined SFV-host co-evolutionary model (see panel **b**). A well supported linear correlation was found (linear regression: N = 16, R^2^ = 0.8032, p < 0.0001), represented by a *solid black line*. The *dots* are labelled with *roman numerals* (I–XVI), referring to branches in Additional file 1: Figure S1

As discussed above, many of the inferred co-divergence events cannot be placed conclusively onto the trees. To further evaluate the co-speciation events and to determine which alternative scenarios are more likely, we time-calibrated the SFV tree using the dates of some of the co-speciation events that could be mapped conclusively onto the trees, directly inferred from the host timescales estimated in [29]. Interestingly, although our reconciliation analyses suggested that the *Pithecia*-*Cacajao* SFV lineage separation and the *Cebus*-*Callithrix* SFV lineage separation are FV-host co-divergence events, the inferred dates suggested otherwise. While the branching dates of the *Pithecia* and *Cebus* SFV lineages were inferred to be ~13.29, and ~19.95 Ma, respectively, the split of the *Pithecia* SFV lineage was topologically determined to be before that of the *Cebus* SFV lineage with strong support (posterior probability = 0.89, Fig. 3b; Additional file 1: Figure S1). This implies that at least one of these two SFV splits was incorrectly determined as a co-speciation event, and therefore could not be used to calibrate the tree. In total, 4 dates were used for time-calibration (Table 5): (1) the separation date of the NWM-OMWA FVs [~43.47 Ma (95 % HPD = 38.55–48.36), [30]], (2) the branching date of the *Cebus xanthosternos* SFV lineage [~1.95 Ma (95 % HPD = 0.91–3.31), [30]], (3) the branching date of the *Ateles chamek* SFV lineage [~5.07 Ma (95 % HPD = 2.87–7.50), [30]], and (4) the separation date between the *Alouatta belzebul* and *Alouatta sara* SFV lineages [~4.95 Ma (95 % HPD = 2.93–7.26), [30]]. The time-calibrated SFV tree is shown in Fig. 4a.

**Table 5:** Time to most recent common ancestor (tMRCA) mean estimates for Haplorrhini and simian foamy virus (SFV) polymerase (*pol)* sequences

Branch node	tMRCA SFV *pol*	tMRCA simian phylogeny^a^	Fossil estimate^a^
Simiiformes	39.85 (34.90–44.89)	43.47 (38.55–48.36)^b^	43 ± 4.5
Platyrrhini	22.31 (14.84–31.04)	24.82 (20.55–29.25)	23.5 ± 3.0
*Atelidae* (*Ateles*/*Alouatta* split)	10.77 (7.45–15.04)	16.13 (10.52–21.35)	NA^c^
*Atelidae* (*Ateles*/*Lagothrix* split)	10.77 (7.45–15.04)	11.25 (7.25-15.46)	NA
*Ateles*	6.28 (4.56–8.05)	5.07 (2.87–7.50)^b^	NA
*Alouatta* (excluding *Alouatta palliatta*)	5.54 (3.99–7.27)	4.94 (2.93–7.26)^b^	NA
*Cebus* (*C. apella*/*C. xanthosternos* split)	3.45 (2.61–4.35)	1.95 (0.91–3.31)^b^	NA
*Cebus/Callithrix*	5.20 (3.08–6.06)	19.95 (15.66–24.03)	NA
*Pitheciiinae*	8.11 (4.88–12.33)	13.69 (9.24–18.34)	NA
*Saimirinae* ^d^	16.89 (10.18–24.93)	2.24 (1.05–3.73)	NA

Using an alignment of 412 nt for 74 SFV taxa. Millions of years (MY) ago. Medians inferred using Bayesian methods and a relaxed molecular clock; ranges in parentheses are 95 % highest posterior density intervals

^a^Dating and fossil estimates from Perelman et al. [30]

^b^Dates used to calibrate the SFV tree

^c^NA, not available

^d^Analysis excluded SFV_Sbo4587 which had an unusually long branch length

Overall, our analysis estimated the times to most recent common ancestors (tMRCAs) of SFVs to be comparable to those of their hosts (Table 5), and the nucleotide substitution rate was calculated to be ~2.14 × 10^−8^ (95 % HPD = 1.72 × 10^−8^–2.62 × 10^−8^) substitutions per site per year (s/n/y), which is also similar to previously estimated rates of SFV evolution (~1.7 × 10^−8^ s/n/y [22] and ~ 7.79 × 10^−9^ s/n/y [28]). The estimated date for the separation of the *Pithecia* and *Cacajao* SFVs [~8.11 Ma (95 % HPD = 4.88–12.33)] was found to be relatively more comparable to that of their hosts [~13.69 Ma (95 % HPD = 9.24–18.34), [30]] than that of the *Callithrix* and *Cebus* SFVs [~4.36 Ma (95 % HPD = 3.08–6.06)] which was estimated to happen much later than the host split [~19.95 Ma (95 % HPD = 15.66–24.03), [30]]. These findings thus suggest that the *Pithecia*-*Cacajao* SFV separation is likely a co-speciation event as initially inferred, and the split between the *Cebus* and *Callithrix* SFVs is not, but rather represents a cross-genus transmission between the two host monkeys (Fig. 4b, short blue or green large arrows). These results also imply that the initially inferred cross-genus transmission from an ancestral *Cacajao* monkey to the *Cebus* and *Callithrix* MRCA (Fig. 3c) was erroneous; it is either a cross-genus transmission from an ancestral *Cacajao* to an ancestral *Callithrix* monkey (Fig. 4b, long blue large arrow), or to an ancestral *Cebus* monkey (Fig. 4b, long green large arrow). To distinguish between these two competing alternative scenarios would require additional SFV sequence data from other NWMs, such as *Mico*, *Cebuella*, and *Callimico* monkeys.

Our phylogenetic reconciliation analyses also suggested that the branching of the *Ateles* SFV lineage is an FV-host co-speciation event, but it could be mapped either to the *Ateles*-*Lagothrix* monkey split event (Fig. 3c1) or to the *Ateles*-*Alouatta* monkey split event (Fig. 3c2). Our additional analyses estimated the evolutionary timescale of *Atelidae* SFVs to be ~10.77 Myr old (95 % HPD = 7.45–15.04). This is comparable to the separation date of the *Ateles* and *Lagothrix* monkeys [~11.25 Ma (95 % HPD = 7.25–15.46), [30]], suggesting that the former scenario is more likely.

Lastly, our analyses estimated the evolutionary timescale of *Saimiri* SFV radiation [~16.89 Ma (95 % HPD = 10.18–24.93) to be ~7.5× that of their hosts (~2.24 Ma (95 % HPD = 1.05–3.73), [30]]. This finding suggests that the *Saimiri* SFV clade likely contains one, or more, NWM SFVs lineages that arose from a cross-species transmission event, but for which the ancestral SFV lineages were not sampled. Assuming a stable FV-host co-speciation history, we hypothesized that the ancestral FV ‘ghost lineage’ may have been an *Aotus* SFV (Fig. 4b), since the split between *Aotus* and *Saimiri* monkeys were inferred to occur ~19.95 Ma (95 % HPD = 15.66–24.03) [29], comparable to our estimated *Saimiri* SFV evolutionary timescale. To further examine this hypothesis, *Aotus* SFV sequences are required. However, while several *Aotus* species have been identified with antibodies to NWM SFV antigens, SFV sequences have not been reported from these seropositive animals for phylogenetic analysis to test this hypothesis (Table 4; [28]). Furthermore, we also found that Saimiri SFVs form a separate, ancestral lineage to all other NWM SFVs (Figs. 3, 4a), and that the two diverged from one another ~22.31 Ma (95 % HPD = 14.84–31.04), comparable to the date of the basal NWM diversification [~24.82 Ma (95 % HPD = 20.55–29.25), [30]]. This date in turn supports the hypothesis that the split between *Saimiri* SFVs and the rest is likely a co-speciation event, corresponding to the basal radiation of NWMs. The refined SFV-host co-evolutionary history is shown in Fig. 4b.

An SFV-host divergence correlation analysis (Fig. 4c) was also performed to further evaluate our refined model of SFV-host co-evolution. Unlike the phylogenetic reconciliation analyses, this analysis takes both the topologies of the virus and host trees as well as their branch lengths into account. In this analysis, we identified SFV-host co-diverging branches based on the refined model, and examined if the genetic divergences of the two are linearly correlated. We found that, given the model, the SFV genetic divergence is proportional to that of their hosts (linear regression: R^2^ = 0.8032; *p* < 0.0001, Fig. 4c). This indicates that the divergence of SFVs and their hosts are internally consistent, and simultaneously supporting our model as well as the stable NWM SFV-host co-speciation hypothesis. We note that the number of co-speciation events is reduced by one, and the number of cross-species transmission events is increased by one under the refined model. Nonetheless, these changes do not alter the conclusion about the stable co-speciation history, as the probability for the observed 11 co-speciation events by chance is still significant (*p* = 0.008). Our results extend those of others demonstrating an ancient coevolution of OWMA SFV to include co-speciation of NWM SFV. However, unlike OWMA SFV that has only rare and relatively recent cross-species transmissions, NWM SFVs may have had at least four ancient cross-genus transmission events in their evolutionary history. Sequence analysis of additional NWM SFVs, especially those from *Aotus* species, are required to further evaluate this scenario.

### Zoonotic potential of NWM SFV

The high prevalence and distribution of SFV in many NWM species reported herein and in other studies [28, 29] highlights the potential zoonotic infection risks for persons handling neotropical monkeys in captivity as pets or at zoological and research institutions and via hunting and butchering in the wild. An estimated 28,000 NHPs are also reported to be collected in Peru each year [45], most for use in biomedical research [46], but some of the larger NWMs including capuchin, spider, and woolly monkeys are hunted for bushmeat (http://www.careforthewild.com). These activities increase opportunities for zoonotic transmission of SFV [9]. Nonetheless, only a single study has reported evidence of human infection with NWM SFV in 11.6 % seroreactive but PCR negative persons [29]. These findings suggest exposure without infection or nonspecific seroreactivity to the antigen used in their assay. Alternatively, limited validation of the PCR primers used in the testing may have affected the assay sensitivity for detecting divergent NWM SFV in the seroreactive persons. Host restriction may also have contributed to the lack of productive infection in those seroreactive primate workers with Trim5α and APOBEC3 having been shown to have a broad anti-retroviral activity [47] may contribute to preventing productive infection of humans with some NWM SFVs. However, human Trim5α expressed in Cf2Th cells could inhibit only SFVsqu but not SFVspm or SFVmar suggesting a limited viral suppression potential of NWM SFV by human Trim5α [26]. Little is known about the ability of human APOBEC3 to inhibit NWM SFVs, with most work limited to OWMA SFV, but which showed that the SFV Bet protein from a variety of OWMAs can counteract human APOBEC3 activity, suggesting that the Bet of NWM SFVs may have similar neutralizing activity [48–50]. Additional studies of larger numbers of workers and also of persons who hunt NWMs using the new serological and PCR assays described here are required to further evaluate the risk of human infection with neotropical monkey SFVs.

## Conclusions

We demonstrate here, and expand on previous studies in Brazil, a broad range of neotropical primate species harboring SFV in captive animals from the US and Peru and in wild-caught NWMS from Peru. We identified at least seven novel and divergent SFVs found in *Ateles chamek* (Peruvian spider monkey) and two species of *A. geoffroyi* (black-handed and Mexican spider monkeys), *Cacajao rubicundus* (uakari monkey), *Pithecia pithecia* (saki monkey), *Saimiri sciureus* (squirrel monkey) and *S. boliviensis peruviensis* (Peruvian squirrel monkey), and *Lagothrix lagotricha* (brown wooly monkey). Co-speciation between SFV and their hosts in all three NWM families was inferred but included ancient cross-genus transmissions of SFV from Pitheciinae to Atelidae, *Cacajao* to *Cebus*/*Callithrix*, between *Cebus* and *Callithrix*, and *Lagothrix* to *Alouatta*. Our results provide an important future reference for time-calibrating information essential for NWM SFV evolutionary timescale inference, as well as evolutionary insight of NWM SFVs. Further studies are needed to expand our understanding of the evolutionary history of broader NWM SFVs and to clarify the possible “ghost” lineage identified by our analyses. Furthermore, the new serological and molecular tools reported here will facilitate an assessment of the risk of zoonotic SFV infection in persons naturally or occupationally exposed to NWMs.

## Additional files

10.1186/s12977-015-0214-0 Co-speciation history of New World monkeys (NWM) and simian foamy viruses (SFVs). A consensus NWM tree (left) estimated from a cytochrome-B nucleotide alignment (156 sequences, 618 nt) is compared to a consensus NWM SFV tree (right) estimated from a polymerase nucleotide alignment (80 sequences, 412 nt). Both trees were constructed under the Bayesian phylogenetic framework by using MrBayes 3.2.1 [38], and molecular clocks were not imposed. The host tree was rooted according to the tree in [30]. The FV tree was rooted with ape and Old World monkey SFVs. The ‘backbone’ polymerase sequences in the SFV tree are labelled with ‘**#**’. The scale bars are in the units of substitutions per site, and numbers on nodes are posterior probabilities. Grey lines indicate SFV-host associations. SFVs without associating hosts are labelled in grey, and were excluded from the phylogenetic reconciliation analysis. Thick branches are co-diverging branches used in the SFV-host divergence correlation analysis, labelled with roman numerals (**I**-**XVI**), referring to dots in Fig. 4C. See Supplementary Table S1 for a complete list of species codes used in the study.
10.1186/s12977-015-0214-0 Three letter species codes used for New World monkeys samples in the study and used to label taxa in the phylogenetic analyses (Figs. 3, 4, and S1).
