# Supplementary figures and images for: Wide distribution and ancient evolutionary history of simian foamy viruses in New World primates
Source: Retrovirology. 2015 Oct 29;12:89. doi: 10.1186/s12977-015-0214-0 (PMC4627628; doi:10.1186/s12977-015-0214-0)

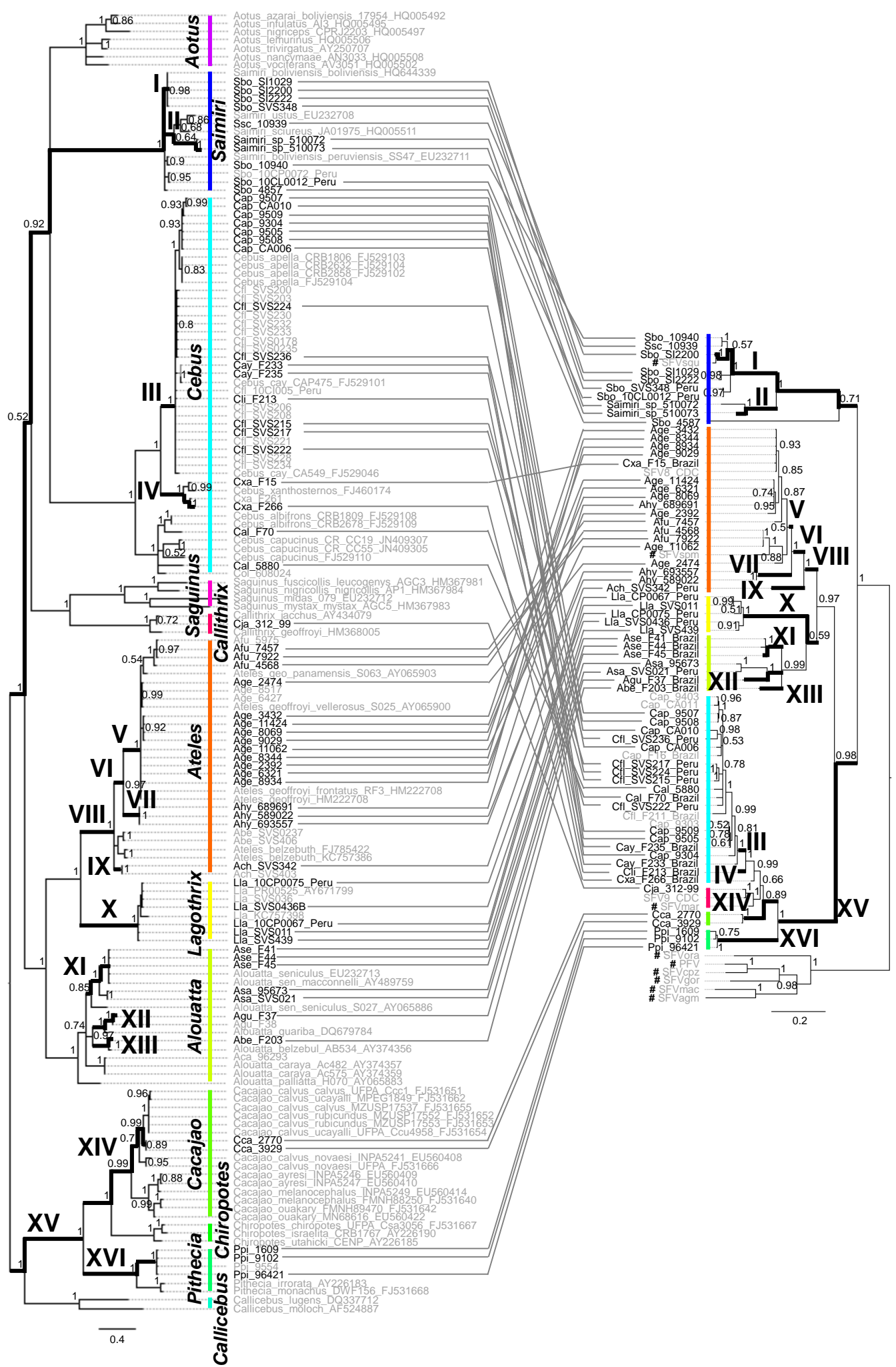

Supplement: Supplementary file 1 — 10.1186/s12977-015-0214-0 Co-speciation history of New World monkeys (NWM) and simian foamy viruses (SFVs). A consensus NWM tree (left) estimated from a cytochrome-B nucleotide alignment (156 sequences, 618 nt) is compared to a consensus NWM SFV tree (right) estimated from a polymerase nucleotide alignment (80 sequences, 412 nt). Both trees were constructed under the Bayesian phylogenetic framework by using MrBayes 3.2.1 [38], and molecular clocks were not imposed. The host tree was rooted according to the tree in [30]. The FV tree was rooted with ape and Old World monkey SFVs. The ‘backbone’ polymerase sequences in the SFV tree are labelled with ‘#’. The scale bars are in the units of substitutions per site, and numbers on nodes are posterior probabilities. Grey lines indicate SFV-host associations. SFVs without associating hosts are labelled in grey, and were excluded from the phylogenetic reconciliation analysis. Thick branches are co-diverging branches used in the SFV-host divergence correlation analysis, labelled with roman numerals (I-XVI), referring to dots in Fig. 4C. See Supplementary Table S1 for a complete list of species codes used in the study. [file 12977_2015_214_MOESM1_ESM.pdf]
