# Supplementary material for: Wide distribution and ancient evolutionary history of simian foamy viruses in New World primates
Source: Retrovirology. 2015 Oct 29;12:89. doi: 10.1186/s12977-015-0214-0 (PMC4627628; doi:10.1186/s12977-015-0214-0)
Supplement: Supplementary file 2 — 10.1186/s12977-015-0214-0 Three letter species codes used for New World monkeys samples in the study and used to label taxa in the phylogenetic analyses (Figs. 3, 4, and S1). [file 12977_2015_214_MOESM2_ESM.docx]

**Table S1.** Three letter species codes used for New World monkeys samples in the study and used to label taxa in the phylogenetic analyses (Figs 3, 4, and S1).

| **Common name**  Black howler monkey | **Scientific name**  *Alouatta caraya* | **Three letter code**  Aca |
| --- | --- | --- |
| Brown howler monkey | *Alouatta guariba* | Agu |
| Red howler  Red howler | *Alouatta seniculus sara*  *Alouatta seniculus* | Asa  Ase |
| Black and red howler | *Alouatta belzebul* | Abe |
| Spider monkey | *Ateles species* | Asp |
| Columbian brown spider | *Ateles belzebuth hybridus* | Ahy |
| Brown-headed spider | *Ateles fusciceps* | Afu |
| Brown-headed spider | *Ateles fusciceps robustus* | Aro |
| Black-handed spider | *Ateles geoffroyi* | Age |
| Mexican spider | *Ateles geoffroyi vellerosus* | Ave |
| Peruvian black spider | *Ateles paniscus chamek* | Ach |
| Red uakari | *Cacajao rubicundus* | Cru |
| Common marmoset | *Callithrix jacchus* | Cja |
| Capuchin | *Cebus* | Csp |
| Tufted capuchin | *Cebus apella* | Cap |
| Hooded capuchin | *Cebus cay* | Cca |
| Yellow-breasted capuchin  White-fronted capuchin | *Cebus xanthosternos*  *Cebus albifrons* | Cxa  Cal |
| Common wooly monkey | *Lagothrix lagotricha* | Lla |
| White-faced saki monkey | *Pithecia pithecia* | Ppi |
| Bolivian squirrel monkey  Peruvian squirrel monkey | *Saimiri boliviensis*  *Saimiri boliviensis peruviensis* | Sbo  Spe |
| Common squirrel | *Saimiri sciureus* | Ssc |
| Squirrel monkey | *Saimiri species* | Ssp |
|  |  |  |
|  |  |  |
|  |  |  |
|  |  |  |
|  |  |  |
|  |  |  |
|  |  |  |
|  |  |  |
|  |  |  |
|  |  |  |
|  |  |  |
|  |  |  |
|  |  |  |
|  |  |  |
|  |  |  |
|  |  |  |
|  |  |  |
|  |  |  |
